# A decision making algorithm for economic growth in the digital economy using CRITIC WASPAS based circular picture fuzzy information

**DOI:** 10.1038/s41598-025-31078-y

**Published:** 2025-12-26

**Authors:** Junguang Gao, Ziyao Lin, Quan Liu

**Affiliations:** 1 Beijing Research Institute of Frontier Science, Beijing, 102488 China; 2 Faculty of Business, Lingnan University, Hong Kong Special Administrative Region, Tuen Mun, 999077 China; 3 Guangzhou College of Commerce, Guangzhou, 511363 Guangdong China

**Keywords:** CRITIC-WASPAS technique, Decision-making, Economic growth in the digital economy, Circular picture fuzzy set, Engineering, Mathematics and computing

## Abstract

In this study, we explore the use of fuzzy set (FS) theory to address uncertainty in evaluating strategies that drive economic growth within the digital economy. Specifically, we employ a circular picture fuzzy (CPF) set (CPFS). As economies become increasingly digital, decision-makers face complex challenges in selecting the most effective strategies for growth, ranging from digital infrastructure investment to innovation support and policy reform. To assess these strategic options, we consider five alternatives, each evaluated against five key attributes. To ensure objectivity in weighing these attributes, we first apply the CRITIC (Criteria Importance Through Intercriteria Correlation) method, which calculates weights based on the contrast intensity and interdependencies among the attributes. Next, we use the WASPAS (Weighted Aggregated Sum Product Assessment) method to rank the alternatives. Furthermore, we develop a decision-making algorithm tailored for multi-attribute group decision-making (MAGDM), integrating the CRITIC-WASPAS approach within the CPFS framework. This model is applied to a hypothetical case study focused on digital economy growth strategies, demonstrating its practical utility in uncertain and complex decision environments. After the evaluation, the second alternative, E-Governance and Digital Policy Reform, emerged as the most optimal strategy for promoting economic growth in the digital economy. To validate the robustness of the proposed approach, we conduct a sensitivity analysis and a comparative evaluation with existing methods. The results confirm that our model provides reliable and effective support for strategic decision-making aimed at fostering economic growth in the digital age.

## Introduction

There are many real-world situations where uncertainty and indeterminacy exist. In these situations, traditional binary logic and crisp set theory cannot perform effective modeling and decision-making. To remedy this, the FS theory presented by Zadeh^1^ in 1965 was the first theory to allow the basic set membership to be degrees between 0 and 1. This was the first theory to allow treatment of vague information to be evaluated in degrees, rather than a yes/no, and thus the application of set theory expanded significantly in control systems, pattern recognition, and artificial intelligence.

Though classical FS theory has been successful, it still combines, somewhat awkwardly, the ideas of membership and non-membership into one scalar value, as well as the lack of and the contradictory presence of evidence. To help remedy this, Atanassov^2^ in 1986 proposed intuitionistic FS (IFS) as a generalization that explicitly models membership and non-membership degrees separately, as long as the sum does not exceed one. This innovation added the dimension of hesitation, which describes the residual uncertainty or the indeterminacy caused by incomplete information. An IFS is, therefore, able to provide a more comprehensive and sophisticated representation of uncertainty, particularly in situations involving decision-making and ambiguity with conflicting information. Adding the abstinence component in IFS makes picture FS (PFS) particularly useful in decision-making problems where stakeholders may not fully agree or disagree and instead express indifference or partial hesitation. PFS offers a richer framework for modeling real-world human opinions. Cuong^3^ in 2013, extended the concept of IFS by adding the degree of abstinence. In a PFS, each element is characterized by three independent membership degrees.

Despite their effectiveness, conventional IFS imply that the membership and non-membership degrees assigned to elements are precise, point-based values. However, in real-world decision-making scenarios such as risk assessment, medical diagnosis, or strategic planning, decision-makers (ℰ) often provide estimates with a degree of uncertainty or reluctance that is not adequately represented by a predefined pair of membership and non-membership degrees. To address this limitation, circular IFS (CIFS) were introduced by Atanassov^4^ in 2020 as an innovative generalization of IFS. CPFS extends the classical structure by incorporating a radius of uncertainty around the membership and non-membership pair, thereby modeling the possible variation in ℰ opinions. While the case of CPFS discussed by Xu and Zhang^5^ in 2025, the three membership degrees are still present, but instead of being represented as fixed values, they are modeled as a circular region in a three-dimensional membership space. This circular representation allows for the modeling of variability, hesitation, or uncertainty radius around each evaluation point. Conceptually, it means that the actual values of membership, abstinence, and non-membership degrees may vary within a certain range, bounded by the circle. Table 1 summarizes the key differences and features of FS, IFS, PFS, and CPFS:

**Table 1:** Extensions of FS theory.

Theory	Components	Constraint	Feature	Strengths
FS	Membership $\left(\alpha \right)$	$0\le \alpha \le 1$	Allows partial membership	Basic uncertainty
IFS	Membership $\left(\alpha \right)$, non-membership $\left(\delta \right)$	$0\le \alpha +\delta \le 1$	Adds explicit non-membership	Both support and against
PFS	Membership $\left(\alpha \right)$, abstinence $\left(\beta \right)$, non-membership $\left(\delta \right)$	$0\le \alpha +\beta +\delta \le 1$	Introduced abstinence	By adding indifference
CIFS	$\left(\alpha ,\delta \right)$, with radius $\left(\gamma \right)$	$0\le \alpha +\delta \le 1$(center)	Circular region around $\left(\alpha ,\delta \right)$	Directional uncertainty
CPFS	$\left(\alpha ,\beta ,\delta \right)$, with radius $\left(\gamma \right)$	$0\le \alpha +\beta +\delta \le 1$(center)	Circular region around $\left(\alpha ,\beta ,\delta \right)$	Most expressive direction

### Problem statement

Developing countries struggle to identify which industries would profit most from investment, even if the digital economy has the potential to support equitable economic advancement. When resources are limited, it’s important to choose sustainable solutions, can last long and provide good returns. The digital economy is changing global markets as productivity, innovation, and connectivity grow rapidly. For developing countries, joining this digital economy brings both opportunities and challenges. Digital technologies can support growth, but problems like weak infrastructure, not enough skilled workers, and poor regulations can slow progress. The shift toward a digital economy is changing how countries interact with citizens and markets, deliver services, and create economic value. Fast internet, mobile networks, cloud computing, online shopping, artificial intelligence, and digital finance are examples of technologies that are assisting nations in becoming more inventive, productive, and job-creating.

These elements strongly correspond with the tenets of FS theory and are frequently characterized by complexity and uncertainty. Strong tools for assessing such fuzzy notions are provided by the MADM and MAGDM techniques, which allow the integration of several, frequently incompatible evaluation criteria. Recently, researchers have made substantial contributions to this domain, introducing innovative models and techniques to enhance decision-making under uncertainty. One we may refer to, diffusion and economic growth fuzzy intelligent system developed by Liu and Zhao^6^, about reinforcing competitive economy of smart cities in Fuzzy-AHP approach suggested by Subkhan et al.^7^, the role of fuzzy logic in the digital transformation of economics was elaborated by Imamguluyev^8^, and enhancing the performance of vocational education in the digital economy with the application of fuzzy logic algorithm was adopted by Zhang and Wang^9^**.**

### Novelty and advantages

This research introduces a novel hybrid decision-making framework, CRITIC and WASPAS techniques within the CPFS environment. The proposed approach is unique in several key aspects that distinguish it from existing MAGDM models in both theory and practice.

1. To the best of our knowledge, this is the first study to fuse the CRITIC-WASPAS method in the framework of CPFS. Existing works have applied CRITIC-WASPAS in classical, fuzzy, IFS, and PFS environments, but none have addressed decision-making under the CPFS, which offers a more advanced and expressive representation of uncertainty.
2. The suggested framework is generalized; it is possible to see conventional FS, IFS, and even PFS as special cases of the CPFS model under certain conditions (e.g., setting the radius to zero). This generalization increases the model’s flexibility and scalability by enabling it to be used for a variety of decision-making issues.
3. The application of the CRITIC technique guarantees that attribute weights are determined from the data itself, based on contrast strength and conflict across attributes, rather than being awarded subjectively. The WASPAS evaluates the normalized DM and demonstrates the ranking of alternatives.
4. The proposed method is not just theoretical, but it has also been tested on a real-world case study. This proves that the proposed work can easily evaluate the complex options with many interconnected factors and the uncertain opinion of an expert.
5. Sensitivity analysis confirms that the model is reliable, while comparison with traditional models shows the performance accuracy and consistency.

### Contribution

In this study, we extend the CRITIC method and WASPAS method within a CPFS environment. This integration helps capture uncertainty more effectively, especially in situations where experts are unsure, disagree, or provide inputs with specific directions. To evaluate the importance of different attributes and the performance of each alternative, we use linguistic terms expressed inform of CPFVs. The CRITIC method is applied to calculate objective weights for each attribute. This ensures that the weights account for both the variation within each attribute and the level of disagreement among experts.

Next, a modified WASPAS method combining both additive and multiplicative approaches is utilized to integrate the decision matrix (DM). The approach is evaluated by choosing the optimal alternatives for economic expansion. Sensitivity analysis demonstrates that the model remains stable when various parameters are changed. Comparative analysis demonstrates that this hybrid approach handles uncertainty, flexibility, and discriminating power more effectively than current fuzzy decision-making models. It is interesting to note that the hybrid models can be viewed as specific instances of this approach, demonstrating its general applicability to address the challenges of real-life decision-making scenarios.

### Layout

This paper is organized as follows: Section “Introduction” has an introduction to the manuscript, while Section “Literature study” has some related literature reviews. The concept of the CPF framework and its related definitions are presented in Section “Preliminaries”. Section “algorithm of multi-attribute group decision-making” extends the CRITIC-WASPAS algorithm for handling MAGDM problems inside the CPF framework. Section “Case study” presents a real-world case study, while Section “Sensitivity and computational analysis” presents sensitivity analysis. section “Result discussion and comparison” contrasts the suggested model with previous, well-established research, and Section “Conclusion” summarizes the proposed work, discusses the limitations, and offers recommendations for further study.

## Literature study

Decision-making involves selecting from multiple alternatives by evaluating options and weighing outcomes. The MADM approach helps ℰ navigate complex scenarios with multiple objectives, identify trade-offs, assess risks, and optimize outcomes, enhancing decision quality and reliability. Kaur et al.^10^ discussed the selection of a vendor using the analytical hierarchy process based on fuzzy preference programming, while the selection of a vendor based on intuitionistic fuzzy analytical hierarchy process is adopted in^11^. Hussain et al.^12^ initiated the Aczel-Alsina aggregation operator in MADM. Wang et al.^13^ developed MADM picture fuzzy Muirhead mean operators. Kaur et al.^14^ suggested a Pythagorean fuzzy approach for sustainable supplier selection using TODIM; on the other hand, the selection of inventory policy under a Pythagorean fuzzy environment is presented in^15^. Seikh et al.^16^ introduced an aggregation operator based on Frank t-norm and t-conorm: Application to MADM Process. MAGDM extends MADM by involving a group of ℰ who collectively evaluate alternatives across multiple attributes. It is useful in complex evaluations, like economic growth in the digital economy assessments, and suits contexts with diverse stakeholders and multiple attributes. Hussain et al.^17^ elaborate on the TOPSIS approach for MAGDM. Abbas et al.^18^ suggested MAGDM in the MAIRCA approach. Wang^19^ adopted the MAGDM model based on the improved ARAS method.

This article extends the WASPAS technique to the CPF framework, enhancing its applicability in MAGDM. The WASPAS technique, introduced by Zavadskas et al.^20^, combines the weighted sum model (WSM) and the weighted product model (WPM) for comprehensive evaluations. It is a well-liked approach to decision-making that takes into account both qualitative and quantitative aspects, enabling methodical evaluation and prioritization. Using CPF’s capacity to manage uncertainty, we use this expanded method to assess the problem of economic growth in the digital economy. The WASPAS methodology frameworks are compiled in Table 2.

**Table 2:** WASPAS method in earlier publications.

Authors	Year	Method	Application
Rani et al.^21^	(2020)	WASPAS	Physician selection problem
Ayyildiz et al.^22^	(2021)	AHP-WASPAS	Refugee camp
Senapati et al.^23^	(2022)	WASPAS	Illustrative example
Albaity et al.^24^	(2023)	WASPAS	Machine learning
Abbas et al.^25^	(2024)	WASPAS	Medical field
initiated work	(2025)	CRITIC-WASPAS	Economic growth in the digital economy

Attributes are rarely autonomous in real-world decision-making situations; instead, they frequently show intricate linkages and have an impact on one another. Enhancing the precision and dependability of MADM procedures requires identifying and taking these dependencies into consideration. Recent studies have increasingly concentrated on adding attribute interdependencies to assessment models in order to overcome this. The WASPAS method is unique among the current MADM methodologies since it can assess both cost and benefit aspects and has a flexible structure for managing related alternatives. However, as Table 2 illustrates, one major drawback of conventional WASPAS systems is their inability to accept CPFS data.

### Research gap

Traditional FS decision-making models, like FS, IFS, and PFS, are widely used in MAGDM. But they often fail to capture real-world uncertainties such as preference direction, hesitation, or conflicting expert opinions. Furthermore, in this context, objective weighting techniques like CRITIC and WASPAS are lacking. A robust, adaptable, and intelligible MAGDM model that combines CRITIC and WASPAS with CPFS is required to make better decisions under uncertainty:

1. How can the CRITIC method be adapted to compute objective weights within the CPFS framework?
2. What modifications are required for the WASPAS model to handle CPFVs effectively?
3. Does the integration of CRITIC and WASPAS within CPFS improve decision-making accuracy as compared to traditional fuzzy MAGDM approaches?
4. How does the proposed CRITIC-WASPAS-CPFS model perform in terms of stability and robustness under varying uncertainty levels?
5. Does increased internet access directly correlate with GDP growth in low-income countries?
6. What is the causal relationship between digital payment adoption and small business productivity?
7. How does the digital economy contribute differently to economic growth in developed vs. developing nations?

To address these gaps and some research questions, this article proposes a novel extension of the CRITIC-WASPAS model in the framework of CPFS and suggests a methodology for how we can easily solve this.

## Preliminaries

### Definition 1

Ref^4^.**:** A CIFS $A$ on the fixed set $\mathcal{U}$ is if the form

$$A=\left\{\left(\alpha \left(\chi \right),\delta \left(\chi \right);\gamma \left(\chi \right)\right)|\chi \in \mathcal{U}\right\}$$

with the condition: $0\le \alpha \left(\chi \right)+\delta \left(\chi \right)\le 1$ of $\chi \in \mathcal{U}$, where $\alpha \left(\chi \right)\in \left[\mathrm{0,1}\right]$ is the membership degree, $\delta \left(\chi \right)\in \left[\mathrm{0,1}\right]$ is the non-membership degree, and $\gamma \left(\chi \right)\in \left[\mathrm{0,1}\right]$ is the radius of the circle. Additionally, $\mathcal{F}\left(\chi \right)=1-\alpha \left(\chi \right)-\delta \left(\chi \right)$ is said to be an indeterminacy degree.

### Definition 2

Ref^3^.**:** A PFS $A$ on the fixed set $\mathcal{U}$ is of the form

$$A=\left\{\left(\alpha \left(\chi \right),\beta \left(\chi \right),\delta \left(\chi \right);\gamma \left(\chi \right)\right)|\chi \in \mathcal{U}\right\}$$

with the condition: $0\le \alpha \left(\chi \right)+\beta \left(\chi \right)+\delta \left(\chi \right)\le 1$ of $\chi \in \mathcal{U}$, where $\alpha \left(\chi \right)\in \left[\mathrm{0,1}\right]$ is the membership degree, $\beta \left(\chi \right)\in \left[\mathrm{0,1}\right]$ is the abstinence degree, and $\delta \left(\chi \right)\in \left[\mathrm{0,1}\right]$ is the non-membership degree. Additionally, $\mathcal{F}\left(\chi \right)=1-\alpha \left(\chi \right)-\beta \left(\chi \right)-\delta \left(\chi \right)$ is said to be a refusal degree.

### Definition 3

Ref^5^.**:** A CPFS $A$ on the fixed set $\mathcal{U}$ is of the form

$$A=\left\{\left(\alpha \left(\chi \right),\beta \left(\chi \right),\delta \left(\chi \right);\gamma \left(\chi \right)\right)|\chi \in \mathcal{U}\right\}$$

with the condition: $0\le \alpha \left(\chi \right)+\beta \left(\chi \right)+\delta \left(\chi \right)\le 1$ of $\chi \in \mathcal{U}$, where $\alpha \left(\chi \right)\in \left[\mathrm{0,1}\right]$ is the membership degree, $\beta \left(\chi \right)\in \left[\mathrm{0,1}\right]$ is the abstinence degree, $\delta \left(\chi \right)\in \left[\mathrm{0,1}\right]$ is the non-membership degree, and $\gamma \left(\chi \right)\in \left[\mathrm{0,1}\right]$ is the radius of the circle. Additionally, $\mathcal{F}\left(\chi \right)=1-\alpha \left(\chi \right)-\beta \left(\chi \right)-\delta \left(\chi \right)$ is said to be the refusal degree, and the CPF values (CPFVs) are denoted by ${A}_{\mathcal{j}}=({\alpha }_{\mathcal{j}},{\beta }_{\mathcal{j}},{\delta }_{\mathcal{j}},{\gamma }_{\mathcal{j}})$; $\left(\mathcal{j}=\mathrm{1,2},\dots ,\mathcal{n}\right)$.

### Definition 4

Ref^5^.**:** Assume that $A=(\alpha ,\beta ,\delta ,\gamma )$ be the CPF value (CPFV). Then, the score function, denoted by $({\d{\rm S}})$ and the accuracy function, denoted by $({\d{\rm A}}$) is defined as:

1 $${\d{\rm S}}\left(A\right)=\frac{1}{3}\left(\alpha +1-\beta +1-\delta \right)\times \gamma \in \left[-\mathrm{1,1}\right]$$

2 $${\d{\rm A}}\left(A\right)=\frac{1}{3}\left(\alpha +1+\beta +1+\delta \right)\times \gamma \in \left[\mathrm{0,1}\right]$$

### Definition 5

Ref^5^.**:** Any two CPFVs ${A}_{1}=\left({\alpha }_{1},{\beta }_{1},{\delta }_{1},{\gamma }_{1}\right)$ and ${A}_{2}=\left({\alpha }_{2},{\beta }_{2},{\delta }_{2},{\gamma }_{2}\right)$ will satisfied the following results:

3 $${A}_{1}\oplus{A}_{2}=\left({\alpha }_{1}+{\alpha }_{2}-{\alpha }_{1}{\alpha }_{2},{\beta }_{1}{\beta }_{2}\begin{array}{c},{\delta }_{1}{\delta }_{2};{\gamma }_{1}{\gamma }_{2}\end{array}\right)$$

4 $${A}_{1}\otimes{A}_{2}=\left(\begin{array}{c}{\alpha }_{1}{\alpha }_{2},{\beta }_{1}{\beta }_{2}\begin{array}{c},{\delta }_{1}+{\delta }_{2}-{\delta }_{1}{\delta }_{2};{\gamma }_{1}{\gamma }_{2}\end{array}\end{array}\right)$$

5 $$\kappa {A}_{1}=\left(\begin{array}{c}1-{\left(1-{\alpha }_{1}\right)}^{ \kappa },{\left({\beta }_{1}\right)}^{ \kappa },{\left({\delta }_{1}\right)}^{ \kappa }; {\left({\gamma }_{1}\right)}^{ \kappa }\end{array}\right)$$

6 $${\left({A}_{1}\right)}^{ \kappa }=\left(\begin{array}{c}{\left({\alpha }_{1}\right)}^{ \kappa },{\left({\beta }_{1}\right)}^{ \kappa },1-{\left(1-{\delta }_{1}\right)}^{ \kappa };{\left({\gamma }_{1}\right)}^{ \kappa }\end{array}\right)$$

7 $${\left({A}_{1}\right)}^{c}=\left({\delta }_{1},{\beta }_{1},{\alpha }_{1},{\gamma }_{1}\right)$$

### Definition 6

Ref^5^.**:** The CPF weighted geometric (CPFWG) operator for CPFVs ${A}_{\mathcal{j}}\left(\mathcal{j}=\mathrm{1,2},3,\dots ,\mathcal{n}\right)$ is of the form:

$$CPFWG\left({A}_{1},{A}_{2},\dots ,{A}_{\mathcal{j}}\right)=\sum_{\mathcal{j}=1}^{\mathcal{n}}{A}_{\mathcal{j}}{\omega }_{\mathcal{j}}$$

with the condition: ${\omega }_{ \mathcal{j} }\ge 0$ such that $\left(\mathcal{j}=\mathrm{1,2},3,\dots ,\mathcal{n}\right)$ and $\sum {\omega }_{ \mathcal{j} }=1$. Then, the CPFWG operator is expressed as follows:

8 $$CPFWG\left({A}_{1},{A}_{2},\dots ,{A}_{\mathcal{j}}\right)=\left(\prod_{\mathcal{j}=1}^{\mathcal{n}}{{\alpha }_{\mathcal{j}}}^{{\omega }_{\mathcal{j}}},\prod_{\mathcal{j}=1}^{\mathcal{n}}{{\beta }_{\mathcal{j}}}^{{\omega }_{\mathcal{j}}},1-\prod_{\mathcal{j}=1}^{\mathcal{n}}{\left(1-{\delta }_{\mathcal{j}}\right)}^{{\omega }_{\mathcal{j}}},\prod_{\mathcal{j}=1}^{\mathcal{n}}{{\gamma }_{\mathcal{j}}}^{{\omega }_{\mathcal{j}}}\right)$$

## Algorithm of multi-attribute group decision-making

We suggested the linguistic term in the framework of CPFS, stored in Table 3, for the evaluation of alternatives.

**Table 3:** Linguistic terms in the framework of CPFS.

Linguistic term	Membership degree	Abstinence degree	Non-membership degree	Radius
Superior (SUP)	1.00	0.00	0.00	0.33
Dominant (DOM)	0.86	0.04	0.06	0.32
Optimal (OPT)	0.72	0.08	0.12	0.31
Extensive (EXT)	0.50	0.16	0.24	0.30
Considerable (CON)	0.32	0.24	0.32	0.29
Moderate (MOD)	0.22	0.30	0.46	0.33
Nominal (NOM)	0.10	0.15	0.56	0.27
Minimal (MIN)	0.05	0.10	0.70	0.28
Negligible (NEG)	0.00	0.05	0.88	0.31

### Methodology of the CRITIC-WASPAS model based on circular picture fuzzy set

This subsection introduces the CRITIC-WASPAS method, a hybrid decision-making approach that integrates the CRITIC technique for objective weight determination with the WASPAS method for comprehensive evaluation of alternatives. The CRITIC method assigns weights by analyzing the correlation and standard deviation of attributes, capturing both the intensity of contrast and the level of conflict among them. Meanwhile, the WASPAS technique combines the strengths of the WSM and the WPM to evaluate alternatives more effectively.

When extended within the CPFS framework, this method becomes especially powerful in handling uncertainty and imprecise information, making it a reliable tool for capturing expert opinions and supporting informed decision-making. Its versatility makes it applicable across a range of domains, including engineering, management, and environmental sciences. The step-by-step procedure of the proposed hybrid model is detailed below:

**Step 1:** The $kth$ ℰ investigate the DM of CPF information to evaluate alternatives against attributes, utilizing the linguistic terms outlined in Table 3.

9 $${{M}^{k}}_{\mathcal{m}\times \mathcal{n}}=\begin{array}{c}\mathrm{\AA}_{1}\\ \mathrm{\AA}_{2}\\ \vdots \\ \mathrm{\AA}_{\mathcal{m}}\end{array} \left[\begin{array}{cccc}{\mathcal{c}}_{1}& {\mathcal{c}}_{2}& \dots & {\mathcal{c}}_{\mathcal{n}}\\ {{\chi }^{k}}_{11}& {{\chi }^{k}}_{12}& \dots & {{\chi }^{k}}_{1\mathcal{n}}\\ {{\chi }^{k}}_{21}& {{\chi }^{k}}_{22}& \dots & {{\chi }^{k}}_{2\mathcal{n}}\\ \vdots & \vdots & \ddots & \vdots \\ {{\chi }^{k}}_{\mathcal{m}1}& {{\chi }^{k}}_{\mathcal{m}2}& \dots & {{\chi }^{k}}_{\mathcal{m}\mathcal{n}}\end{array}\right]$$

**Step 2:** The weight of $kth$ ℰ such that $\left(k=\mathrm{1,2},3,\dots ,r\right)$ can be obtained with the help of the score function presented in Eq. ($1$) and then accumulate each ℰ weight into the designated Eq. ($10$), where the summation of $kth$ ℰ weight must be equal to 1.

10 $$\frac{{\sum }_{k}\text{ {\d{\rm S}}}\left({{\chi }^{k}}_{\mathcal{i}\mathcal{j}}\right)}{{\sum }_{k}^{r}\left({\sum }_{k}\text{ {\d{\rm S}}}\left({{\chi }^{k}}_{\mathcal{i}\mathcal{j}}\right)\right)}$$

**Step 3:** Aggregated the DM presented in Eq. ($9$) using the formula presented in Eq. (8) under the presence of weight of $kth$ ℰ, and we get the designated DM presented in Eq. (11) such that $\left(\mathcal{i}=\mathrm{1,2},\dots ,\mathcal{m}\right)$ and $\left(\mathcal{j}=\mathrm{1,2},\dots ,\mathcal{n}\right)$ represent the CPFVs of each alternative $\mathcal{i}$ against each attribute $\mathcal{j}$.

11 $${M}_{\mathcal{m}\times \mathcal{n}}=\begin{array}{c}\mathrm{\AA}_{1}\\ \mathrm{\AA}_{2}\\ \vdots \\ \mathrm{\AA}_{\mathcal{m}}\end{array} \left[\begin{array}{cccc}{\mathcal{c}}_{1}& {\mathcal{c}}_{2}& \dots & {\mathcal{c}}_{\mathcal{n}}\\ {\chi }_{11}& {\chi }_{12}& \dots & {\chi }_{1\mathcal{n}}\\ {\chi }_{21}& {\chi }_{22}& \dots & {\chi }_{2\mathcal{n}}\\ \vdots & \vdots & \ddots & \vdots \\ {\chi }_{\mathcal{m}1}& {\chi }_{\mathcal{m}2}& \dots & {\chi }_{\mathcal{m}\mathcal{n}}\end{array}\right]$$

## Step 4: CRITIC Technique

**Step 4.1:** Calculate the score of each value of aggregated DM presented in Eq. (11) with the help of the provided Eq. (12).

12 $${\d{\rm S}}\left({\chi }_{\mathcal{i}\mathcal{j}}\right)=\frac{1}{3}\left({\alpha }_{\mathcal{i}\mathcal{j}}+1-{\beta }_{\mathcal{i}\mathcal{j}}+1-{\delta }_{\mathcal{i}\mathcal{j}}\right)\times {\gamma }_{\mathcal{i}\mathcal{j}}\in \left[-\mathrm{1,1}\right]$$

**Step 4.2:** Normalize the score values of the benefit attribute and cost attribute into a standard matrix by using the conversion formula designated in Eq. (13), where ${{\d{\rm S}}\left({\chi }_{\mathcal{i}\mathcal{j}}\right)}^{+}=\underset{ \mathcal{i} }{\mathrm{max}}\left({\d{\rm S}}\left({\chi }_{\mathcal{i}\mathcal{j}}\right)\right)$ and ${{\d{\rm S}}\left({\chi }_{\mathcal{i}\mathcal{j}}\right)}^{-}=\underset{ \mathcal{i} }{\mathrm{min}}\left({\d{\rm S}}\left({\chi }_{\mathcal{i}\mathcal{j}}\right)\right)$.

13 $$\widetilde{{\d{\rm S}}\left({\chi }_{\mathcal{i}\mathcal{j}}\right)}=\left\{\begin{array}{c}\frac{{\d{\rm S}}\left({\chi }_{\mathcal{i}\mathcal{j}}\right)-{{\d{\rm S}}\left({\chi }_{\mathcal{i}\mathcal{j}}\right)}^{-}}{{{\d{\rm S}}\left({\chi }_{\mathcal{i}\mathcal{j}}\right)}^{+}-{{\d{\rm S}}\left({\chi }_{\mathcal{i}\mathcal{j}}\right)}^{-}}; \text{for benefit attribute} \\ \frac{{{\d{\rm S}}\left({\chi }_{\mathcal{i}\mathcal{j}}\right)}^{+}-{\d{\rm S}}\left({\chi }_{\mathcal{i}\mathcal{j}}\right)}{{{\d{\rm S}}\left({\chi }_{\mathcal{i}\mathcal{j}}\right)}^{+}-{{\d{\rm S}}\left({\chi }_{\mathcal{i}\mathcal{j}}\right)}^{-}}; \text{for cost attribute}\end{array}\right.$$

**Step 4.3:** Calculate the standard deviations of each attribute with the help of the provided Eq. (14), where $\stackrel{-}{{\d{\rm S}}\left({\chi }_{\mathcal{i}\mathcal{j}}\right)}=\sum_{\mathcal{i}=1}^{\mathcal{m}}\frac{\widetilde{ {\d{\rm S}}\left({\chi }_{\mathcal{i}\mathcal{j}}\right)}}{\mathcal{m}}$.

14 $${\mathfrak{D}}_{\mathcal{j}}=\sqrt{\frac{\sum_{\mathcal{i}=1}^{\mathcal{m}}{\left({\d{\rm S}}\left({\chi }_{\mathcal{i}\mathcal{j}}\right)-\stackrel{-}{{\d{\rm S}}\left({\chi }_{\mathcal{i}\mathcal{j}}\right)}\right)}^{2}}{\mathcal{m}}}$$

**Step 4.4:** Determined the correlation coefficient for the attribute using Eq. (15).

15 $${\mathbb{C}}_{\mathcal{j}\mathfrak{t}}=\frac{\sum_{\mathcal{i}=1}^{\mathcal{m}}\left({\d{\rm S}}\left({\chi }_{\mathcal{i}\mathcal{j}}\right)-\stackrel{-}{{\d{\rm S}}\left({\chi }_{\mathcal{j}}\right)}\right)\left({\d{\rm S}}\left({\chi }_{\mathcal{i}\mathcal{j}}\right)-\stackrel{-}{{\d{\rm S}}\left({\chi }_{\mathfrak{t}}\right)}\right)}{\sqrt{\sum_{\mathcal{i}=1}^{\mathcal{m}}{\left({\d{\rm S}}\left({\chi }_{\mathcal{i}\mathcal{j}}\right)-\stackrel{-}{{\d{\rm S}}\left({\chi }_{\mathcal{j}}\right)}\right)}^{2}\sum_{\mathcal{i}=1}^{\mathcal{m}}{\left({\d{\rm S}}\left({\chi }_{\mathcal{i}\mathcal{j}}\right)-\stackrel{-}{{\d{\rm S}}\left({\chi }_{\mathfrak{t}}\right)}\right)}^{2}}}$$

**Step 4.5:** Accumulate the information for each attribute by using Eq. (16).

16 $${\mathcal{c}}_{\mathcal{j}}={\mathfrak{D}}_{\mathcal{j}}\sum_{\mathcal{j}=1}^{\mathcal{n}}\left(1-{\mathbb{C}}_{\mathcal{j}\mathfrak{t}}\right)$$

**Step 4.6:** The weights prioritization of each attribute is obtained with the help of Eq. (17).

17 $$\widetilde{{\omega }_{\mathcal{j}}}=\frac{{\mathcal{c}}_{\mathcal{j}}}{\sum_{\mathcal{j}=1}^{\mathcal{n}}{\mathcal{c}}_{\mathcal{j}}}$$

## Step 5: WASPAS Method

**Step 5.1:** No need for normalization whenever DM has only benefit attributes. In the case of the cost attribute, we are required to utilize Eq. (18).

18 $$\varphi {\d{\rm S}}\left({\chi }_{\mathcal{i}\mathcal{j}}\right)=\left\{\begin{array}{c}\frac{{\d{\rm S}}\left({\chi }_{\mathcal{i}\mathcal{j}}\right)}{\underset{ \mathcal{i} }{\mathrm{max}}\left({\d{\rm S}}\left({\chi }_{\mathcal{i}\mathcal{j}}\right)\right)}; \text{for benefit attribute} \\ \frac{{\d{\rm S}}\left({\chi }_{\mathcal{i}\mathcal{j}}\right)}{\underset{ \mathcal{i} }{\mathrm{min}}\left({\d{\rm S}}\left({\chi }_{\mathcal{i}\mathcal{j}}\right)\right)}; \text{for cost attribute}\end{array}\right.$$

**Step 5.2:** As per the WASPAS technique, the WSM results are denoted by ${\mathbb{Q}}_{\mathcal{i}}^{1}$ provided with the help of Eq. (19).

19 $${\mathbb{Q}}_{\mathcal{i}}^{1}=\sum_{\mathcal{j}=1}^{\mathcal{n}}\varphi {\d{\rm S}}\left({\chi }_{\mathcal{i}\mathcal{j}}\right)\widetilde{{\omega }_{\mathcal{j}}}$$

**Step 5.3:** As per the WASPAS technique, the WPM results are denoted by ${\mathbb{Q}}_{\mathcal{i}}^{2}$ provided with the help of Eq. (20).

20 $${\mathbb{Q}}_{\mathcal{i}}^{2}=\prod_{\mathcal{j}=1}^{\mathcal{n}}{\varphi {\d{\rm S}}\left({\chi }_{\mathcal{i}\mathcal{j}}\right)}^{\widetilde{{\omega }_{\mathcal{j}}}}$$

**Step 5.4:** WASPAS method denoted by ${\mathbb{Q}}_{\mathcal{i}}$, combines the strengths of the WSM and the WPM, with the help of the convex formula presented in Eq. (21), to enhance ranking accuracy as $\lambda \in \left[\mathrm{0,1}\right]$:

21 $${\mathbb{Q}}_{\mathcal{i}}=\lambda {\mathbb{Q}}_{\mathcal{i}}^{1}+\left(1-\lambda \right){\mathbb{Q}}_{\mathcal{i}}^{2}$$

**Fig. 1:**
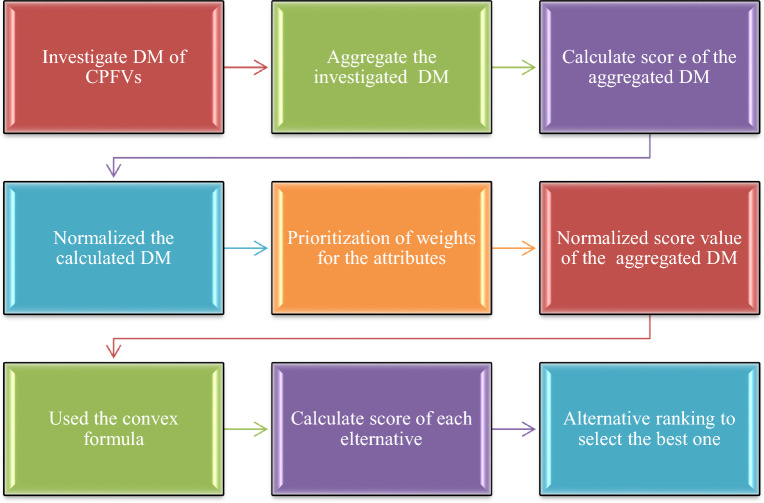
Evaluation of the MAGDM problem on the CRITIC-WASPAS method.

For more clarity, we capture Figure 1, which shows that the MAGDM problems can be easily solved with the help of the hybrid model CRITIC-WASPAS in the CPF framework.

## Case study

This study aims to identify the most effective strategy for promoting economic growth in the digital economy, focusing on developing countries. By comparing five strategic alternatives using five key attributes, the study will provide actionable insights for policymakers and stakeholders.

In this case study, we use the suggested CRITIC-WASPAS technique within the CPF to examine the challenges of promoting economic growth in the digital economy. Utilized the MAGDM technique to evaluate and address the problems associated with the growth of the digital economy.

### Proposed problem alternatives

We examine five different alternatives for economic growth in the digital economy in this context.

- $\textbf{\AA}_{1}$**:** Digital Infrastructure Development
- $\textbf{\AA}_{2}$**:** E-Governance and Digital Policy Reform
- $\textbf{\AA}_{3}$**:** Enhancing Cyber Security and Data Protection Laws
- $\textbf{\AA}_{4}$**:** Digital Skills and Education
- $\textbf{\AA}_{4}$**:** Financial Inclusion via Digital Platforms

### Proposed problem attributes

Assume that each alternative has five unique characteristics to evaluate the alternatives for economic growth in the digital economy.

- $${\mathcal{c}}_{{ }1}$$**:** Scalability
- ${\mathcal{c}}_{\boldsymbol{ }2}$**:** Time to Impact
- $${\mathcal{c}}_{\boldsymbol{ }3}$$: Cost-Effectiveness
- $${\mathcal{c}}_{\boldsymbol{ }4}$$**:** Economic Impact
- $${\mathcal{c}}_{\boldsymbol{ }5}$$**:** Implementation Cost

### Proposed problem evaluation

To address the difficulties of economic growth in the digital economy based on the proposed hybrid model, we consider hypothetical data that were used for this purpose. Our novel CRITIC-WASPAS method successfully handles the underlying complexity. The proposed methodology can be applied to optimally enhance policy and strategic decision-making aimed at accelerating economic growth in the digital era.

**Step 1:** The three ℰ investigate the DM of CPFVs depicted in Table 4, utilizing the linguistic terms presented in Table 3, to evaluate alternatives against attributes.

**Table 4:** The ℰ investigates the linguistic terms for the evaluation of alternatives.

$\mathcal{E}$	$\textbf{\AA}/\mathcal{c}$	${\mathcal{c}}_{1}$	${\mathcal{c}}_{2}$	${\mathcal{c}}_{3}$	${\mathcal{c}}_{4}$	${\mathcal{c}}_{5}$
${\mathcal{E}}_{1}$	$\mathrm{\AA}_{1}$	DOM	CON	NEG	OPT	MIN
	$\mathrm{\AA}_{2}$	EXT	DOM	NEG	DOM	NEG
	$\mathrm{\AA}_{3}$	OPT	EXT	MIN	SUP	MIN
	$\mathrm{\AA}_{4}$	EXT	SUP	NEG	CON	NOM
	$\mathrm{\AA}_{5}$	DOM	EXT	MIN	EXT	NEG
${\mathcal{E}}_{2}$	$\mathrm{\AA}_{1}$	MIN	OPT	NOM	EXT	NEG
	$\mathrm{\AA}_{2}$	OPT	DOM	MIN	DOM	NEG
	$\mathrm{\AA}_{3}$	DOM	EXT	NEG	NEG	MIN
	$\mathrm{\AA}_{4}$	OPT	SUP	MIN	DOM	MIN
	$\mathrm{\AA}_{5}$	DOM	OPT	NEG	DOM	NEG
${\mathcal{E}}_{3}$	$\mathrm{\AA}_{1}$	EXT	OPT	NEG	EXT	MIN
	$\mathrm{\AA}_{2}$	SUP	SUP	NEG	DOM	MIN
	$\mathrm{\AA}_{3}$	OPT	EXT	NEG	SUP	NEG
	$\mathrm{\AA}_{4}$	MOD	DOM	MIN	OPT	NEG
	$\mathrm{\AA}_{5}$	OPT	CON	NEG	EXT	MIN

**Step 2:** To determine the weights of three ℰ linguistic terms from Table 3 are utilized, and Eq. (10) is applied to calculate the weights, which are then presented in Table 5.

**Table 5:** The weights of ℰ to evaluate the alternatives.

${\varvec{k}}{\varvec{t}}{\varvec{h}}$ ℰ	Role	Responsibility	Decision	Weights
${\mathcal{E}}_{1}$	DOM	DOM	MIN	0.37
${\mathcal{E}}_{2}$	SUP	NOM	NOM	0.30
${\mathcal{E}}_{3}$	MIN	CON	SUP	0.33

**Step 3:** Aggregated the DM designated in Table 4 based on Eq. (8), and the obtained results are showcased in Table 6

**Table 6:** Aggregated DM

$\textbf{\AA}/\mathcal{c}$	${\mathcal{c}}_{1}$	${\mathcal{c}}_{2}$	${\mathcal{c}}_{3}$	${\mathcal{c}}_{4}$	${\mathcal{c}}_{5}$
$\textbf{\AA}_{1}$	$$\left(\begin{array}{c}\mathrm{0.31,0.08,0.38},\\ 0.30\end{array}\right)$$	$$\left(\begin{array}{c}\mathrm{0.53,0.12,0.20},\\ 0.30\end{array}\right)$$	$$\left(\begin{array}{c}\mathrm{0.00,0.07,0.82},\\ 0.30\end{array}\right)$$	$$\left(\begin{array}{c}\mathrm{0.57,0.12,0.20},\\ 0.30\end{array}\right)$$	$$\left(\begin{array}{c}\mathrm{0.00,0.08,0.77},\\ 0.29\end{array}\right)$$
$\textbf{\AA}_{2}$	$$\left(\begin{array}{c}\mathrm{0.70,0.00,0.13},\\ 0.31\end{array}\right)$$	$$\left(\begin{array}{c}\mathrm{0.90,0.00,0.04},\\ 0.32\end{array}\right)$$	$$\left(\begin{array}{c}\mathrm{0.00,0.06,0.84},\\ 0.30\end{array}\right)$$	$$\left(\begin{array}{c}\mathrm{0.86,0.04,0.06},\\ 0.32\end{array}\right)$$	$$\left(\begin{array}{c}\mathrm{0.00,0.06,0.84},\\ 0.30\end{array}\right)$$
$\textbf{\AA}_{3}$	$$\left(\begin{array}{c}\mathrm{0.76,0.06,0.10},\\ 0.31\end{array}\right)$$	$$\left(\begin{array}{c}\mathrm{0.50,0.16,0.24},\\ 0.30\end{array}\right)$$	$$\left(\begin{array}{c}\mathrm{0.00,0.06,0.83},\\ 0.30\end{array}\right)$$	$$\left(\begin{array}{c}\mathrm{0.00,0.00,0.47},\\ 0.33\end{array}\right)$$	$$\left(\begin{array}{c}\mathrm{0.00,0.08,0.78},\\ 0.29\end{array}\right)$$
$\textbf{\AA}_{4}$	$$\left(\begin{array}{c}\mathrm{0.43,0.16,0.29},\\ 0.31\end{array}\right)$$	$$\left(\begin{array}{c}\mathrm{0.95,0.00,0.02},\\ 0.33\end{array}\right)$$	$$\left(\begin{array}{c}\mathrm{0.00,0.08,0.79},\\ 0.29\end{array}\right)$$	$$\left(\begin{array}{c}\mathrm{0.56,0.10,0.18},\\ 0.31\end{array}\right)$$	$$\left(\begin{array}{c}\mathrm{0.00,0.09,0.74},\\ 0.29\end{array}\right)$$
$\textbf{\AA}_{4}$	$$\left(\begin{array}{c}\mathrm{0.81,0.05,0.08},\\ 0.32\end{array}\right)$$	$$\left(\begin{array}{c}\mathrm{0.48,0.15,0.23},\\ 0.30\end{array}\right)$$	$$\left(\begin{array}{c}\mathrm{0.00,0.06,0.83},\\ 0.30\end{array}\right)$$	$$\left(\begin{array}{c}\mathrm{0.59,0.11,0.19},\\ 0.31\end{array}\right)$$	$$\left(\begin{array}{c}\mathrm{0.00,0.06,0.84},\\ 0.30\end{array}\right)$$

## Step 4: CRITIC Technique

**Step 4.1:** The score values of aggregate DM (presented in Table 6) are showcased in Table 7; these score values are obtained with the help of Eq. (12).

**Table 7:** Score values of the aggregated DM.

$\textbf{\AA}/\mathcal{c}$	${\mathcal{c}}_{1}$	${\mathcal{c}}_{2}$	${\mathcal{c}}_{3}$	${\mathcal{c}}_{4}$	${\mathcal{c}}_{5}$
$\textbf{\AA}_{1}$	019	0.22	0.11	0.23	0.11
$\textbf{\AA}_{2}$	0.27	0.31	0.11	0.29	0.11
$\textbf{\AA}_{3}$	0.27	0.21	0.11	0.17	0.11
$\textbf{\AA}_{4}$	0.20	0.32	0.11	0.23	0.11
$\textbf{\AA}_{4}$	0.28	0.21	0.11	0.23	0.11

**Step 4.2:** We are required to normalize Table 7, because the classified attributes have both types (first, second, and fourth are benefits, while third and fifth are costs). The normalized DM is obtained with the help of Eq. (13) and presented in Table 8.

**Table 8:** Normalized DM.

$\textbf{\AA}/\mathcal{c}$	${\mathcal{c}}_{1}$	${\mathcal{c}}_{2}$	${\mathcal{c}}_{3}$	${\mathcal{c}}_{4}$	${\mathcal{c}}_{5}$
$\textbf{\AA}_{1}$	0.00	0.12	1.00	0.47	0.00
$\textbf{\AA}_{2}$	0.85	0.89	0.59	1.00	1.00
$\textbf{\AA}_{3}$	0.86	0.00	0.55	0.00	0.14
$\textbf{\AA}_{4}$	0.19	1.00	0.00	0.52	0.11
$\textbf{\AA}_{4}$	1.00	0.00	0.55	0.53	1.00

**Step 4.3:** According to Eq. (14), we obtained the standard deviations of each attribute and presented them in Table 9.

**Table 9:** Standard deviation for each attribute.

Standard deviations	${\mathcal{c}}_{1}$	${\mathcal{c}}_{2}$	${\mathcal{c}}_{3}$	${\mathcal{c}}_{4}$	${\mathcal{c}}_{5}$
${\mathfrak{D}}_{\mathcal{j}}$	0.40	0.45	0.32	0.32	0.45

**Step 4.4:** Between any two attributes, the correlation coefficient is obtained with the help of Eq. (15), and the resulting values are showcased in Table 10.

**Table 10:** Correlation coefficient of each attribute.

${\mathcal{C}}_{\mathcal{j}\mathfrak{t}}$	${\mathcal{c}}_{1}$	${\mathcal{c}}_{2}$	${\mathcal{c}}_{3}$	${\mathcal{c}}_{4}$	${\mathcal{c}}_{5}$
${\mathcal{c}}_{1}$	1.00	−0.23	−0.06	0.03	0.76
${\mathcal{c}}_{2}$	−0.23	1.00	−0.61	0.64	0.10
${\mathcal{c}}_{3}$	−0.06	−0.61	1.00	0.00	0.01
${\mathcal{c}}_{4}$	0.03	0.64	0.00	1.00	0.63
${\mathcal{c}}_{5}$	0.76	0.10	0.01	0.63	1.00

**Step 4.5:** The information of each attribute is obtained by using Eq. (16) and is designated in Table 11.

**Table 11:** Information for each attribute.

Attributes	${\mathcal{c}}_{1}$	${\mathcal{c}}_{2}$	${\mathcal{c}}_{3}$	${\mathcal{c}}_{4}$	${\mathcal{c}}_{5}$
${\mathcal{c}}_{\mathcal{j}}$	1.41	1.84	1.48	0.86	1.13

**Step 4.6:** The prioritized weight for each attribute is obtained by using Eq. (17) and presented in Table 12.

**Table 12:** Prioritized weights for each attribute.

Weights	${\mathcal{c}}_{1}$	${\mathcal{c}}_{2}$	${\mathcal{c}}_{3}$	${\mathcal{c}}_{4}$	${\mathcal{c}}_{5}$
$\widetilde{{{\varvec{\omega}}}_{\mathcal{j}}}$	0.21	0.27	0.22	0.13	0.17

## Step 5: WASPAS Technique

**Step 5.1:** The attributes are classified into benefit attributes (first, second, and fourth) and cost attributes (third and fifth). Based on this classification, we are required to normalize the aggregated DM presented in Table 7 by using Eq. (18), and then the normalized DM is delighted in Table 13**.**

**Table 13:** Normalized DM.

$\textbf{\AA}/\mathcal{c}$	${\mathcal{c}}_{1}$	${\mathcal{c}}_{2}$	${\mathcal{c}}_{3}$	${\mathcal{c}}_{4}$	${\mathcal{c}}_{5}$
$\textbf{\AA}_{1}$	0.66	0.69	1.00	0.77	1.00
$\textbf{\AA}_{2}$	0.95	0.96	1.00	1.00	1.00
$\textbf{\AA}_{3}$	0.95	0.65	1.00	0.56	1.00
$\textbf{\AA}_{4}$	0.73	1.00	1.00	0.79	1.00
$\textbf{\AA}_{4}$	1.00	0.65	1.00	0.79	1.00

**Step 5.2 to Step 5.4:** According to the WASPAS method, when $\lambda =0.5$, the WSM and WPM are combined generalized evaluations are calculated using Eq. (19) to Eq. (21), respectively. The results are summarized in Table 14**.**

**Table 14:** Score values and ranking.

Proposed	$\textbf{\AA}_{1}$	$\textbf{\AA}_{2}$	$\textbf{\AA}_{3}$	$\textbf{\AA}_{4}$	$\textbf{\AA}_{4}$	Ranking result	Optimal
Score of ${{\varvec{Q}}}_{\mathcal{i}}^{1}$	0.82	0.98	0.84	0.92	0.88	$\mathrm{\AA}_{2}>\mathrm{\AA}_{4}>\mathrm{\AA}_{5}>\mathrm{\AA}_{3}>\mathrm{\AA}_{1}$	$\mathrm{\AA}_{2}$
Score of ${{\varvec{Q}}}_{\mathcal{i}}^{2}$	0.80	0.98	0.82	0.91	0.87	$\mathrm{\AA}_{2}>\mathrm{\AA}_{4}>\mathrm{\AA}_{5}>\mathrm{\AA}_{3}>\mathrm{\AA}_{1}$	$\mathrm{\AA}_{2}$
Score of ${{\varvec{Q}}}_{\mathcal{i}}$	0.81	0.98	0.83	0.91	0.87	$\mathrm{\AA}_{2}>\mathrm{\AA}_{4}>\mathrm{\AA}_{5}>\mathrm{\AA}_{3}>\mathrm{\AA}_{1}$	$\mathrm{\AA}_{2}$

The overall ranking of alternatives in Table 14 under different scenarios is given below:

- WSM ranking is $\mathrm{\AA}_{2}>\mathrm{\AA}_{4}>\mathrm{\AA}_{5}>\mathrm{\AA}_{3}>\mathrm{\AA}_{1}$
- WPM ranking is $\mathrm{\AA}_{2}>\mathrm{\AA}_{4}>\mathrm{\AA}_{5}>\mathrm{\AA}_{3}>\mathrm{\AA}_{1}$
- WASPAS ranking is $\mathrm{\AA}_{2}>\mathrm{\AA}_{4}>\mathrm{\AA}_{5}>\mathrm{\AA}_{3}>\mathrm{\AA}_{1}$

**Fig. 2:**
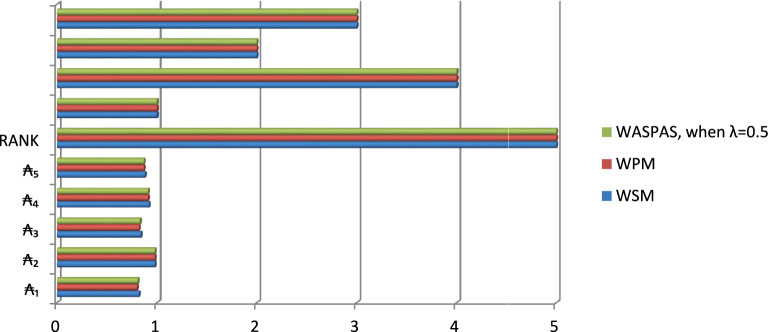
Graphical illustration of score value and ranking of alternatives based on initiated work.

This indicates that, in each case, $\mathrm{\AA}_{2}$ is the optimal choice, which represents the E-Governance and Digital Policy Reform. Figure 2 further illustrates the behavior and ranking of alternatives, providing a clear visual representation of the results.

Figure 2 displays the ranking and score values of each alternative when $\lambda =0.5$, based on the proposed algorithm. The results show that $\textbf{\AA}_{2}$ emerges as the most suitable alternative under both ${\mathbb{Q}}_{\mathcal{i}}^{1}$ and ${\mathbb{Q}}_{\mathcal{i}}^{2}$. Moreover, $\mathrm{\AA}_{2}$ consistently ranks highest under ${\mathbb{Q}}_{\mathcal{i}}$, demonstrating its robustness across different evaluation scenarios.

### Analysis of variance for the validation of ranking result

To validate the robustness of the ranking results obtained through the CRITIC–WASPAS method within the CPFS framework, a one-way Analysis of Variance (ANOVA) was conducted. The purpose of this test is to statistically verify whether significant differences exist among the performance scores of the five strategic alternatives. If the differences in means are statistically significant, then the ranking generated by the proposed MAGDM model can be considered reliable and not driven by random variation. Thus, ANOVA serves as an additional statistical layer to confirm the correctness of the optimal alternative identified in the study.

The ANOVA test was applied to the score values of the aggregated DM presented in Table 7. The results indicate a statistically significant difference among the mean performance levels of the alternatives ($F=4.92$, p=0.007). Since the p-value is below 0.05, the null hypothesis of equal means is rejected. This indicates that the performance levels of the alternatives are not the same, and the ranking produced by the proposed method reflects real differences rather than random variation. The ranking results of alternatives are summarized in Table 15.

**Table 15:** ANOVA-Validated ranking result.

Alternatives	Mean score values	Position of alternatives
$\textbf{\AA}_{1}$	0.17	5
$\textbf{\AA}_{2}$	0.22	1
$\textbf{\AA}_{3}$	0.18	4
$\textbf{\AA}_{4}$	0.19	3
$\textbf{\AA}_{4}$	0.21	2

To further interpret the ANOVA outcome, the mean of the score values of the aggregated DM for each alternative was computed. The results show that alternative $\mathrm{\AA}_{2}$ (E-Governance and Digital Policy Reform) has the highest mean score among all alternatives. This observation is fully consistent with the CRITIC–WASPAS ranking, which also identifies $\mathrm{\AA}_{2}$ as the most effective strategy for digital economic growth. Alternatives $\mathrm{\AA}_{1}$ and $\mathrm{\AA}_{3}$ exhibit the lowest mean scores, while $\mathrm{\AA}_{4}$ and $\mathrm{\AA}_{5}$ demonstrate moderate performance. The statistical evidence, therefore, supports the correctness and reliability of the ranking order and particularly reinforces the optimality of the alternative $\mathrm{\AA}_{2}$.

## Sensitivity and computational analysis

To evaluate the robustness of the decision-making results, sensitivity analysis is conducted within the WASPAS method by varying the balancing parameter $\lambda$, which determines the relative importance of the WSM and the WPM. By systematically changing $\lambda$ values typically from 0 to 1 in increments from 0.1 to 0.9, the combined WASPAS score for each alternative is recalculated, and the corresponding rankings are analyzed. This process helps identify how sensitive the final decision is to changes in the aggregation strategy. If the top-ranked alternative remains consistent across different $\lambda$ values, it indicates a high level of stability and reliability in the decision-making outcome. The strength and consistency of the hybrid CRITIC-WASPAS method are displayed in Table 16.

**Table 16:** Influence the result based on different parameters.

${\varvec{\lambda}}$	$\textbf{\AA}_{1}$	$\textbf{\AA}_{2}$	$\textbf{\AA}_{3}$	$\textbf{\AA}_{4}$	$\textbf{\AA}_{4}$	Ranking
${{\varvec{\lambda}}}_{1}=0.1$	0.80	0.98	0.82	0.91	0.87	$\mathrm{\AA}_{2}>\mathrm{\AA}_{4}>\mathrm{\AA}_{5}>\mathrm{\AA}_{3}>\mathrm{\AA}_{1}$
${{\varvec{\lambda}}}_{2}=0.2$	0.81	0.98	0.82	0.91	0.87	$\mathrm{\AA}_{2}>\mathrm{\AA}_{4}>\mathrm{\AA}_{5}>\mathrm{\AA}_{3}>\mathrm{\AA}_{1}$
${{\varvec{\lambda}}}_{3}=0.3$	0.81	0.98	0.83	0.91	0.87	$\mathrm{\AA}_{2}>\mathrm{\AA}_{4}>\mathrm{\AA}_{5}>\mathrm{\AA}_{3}>\mathrm{\AA}_{1}$
${{\varvec{\lambda}}}_{4}=0.4$	0.81	0.98	0.83	0.91	0.87	$\mathrm{\AA}_{2}>\mathrm{\AA}_{4}>\mathrm{\AA}_{5}>\mathrm{\AA}_{3}>\mathrm{\AA}_{1}$
${{\varvec{\lambda}}}_{5}=0.5$	0.81	0.98	0.83	0.91	0.87	$\mathrm{\AA}_{2}>\mathrm{\AA}_{4}>\mathrm{\AA}_{5}>\mathrm{\AA}_{3}>\mathrm{\AA}_{1}$
${{\varvec{\lambda}}}_{6}=0.6$	0.81	0.98	0.83	0.91	0.87	$\mathrm{\AA}_{2}>\mathrm{\AA}_{4}>\mathrm{\AA}_{5}>\mathrm{\AA}_{3}>\mathrm{\AA}_{1}$
${{\varvec{\lambda}}}_{7}=0.7$	0.81	0.98	0.84	0.92	0.88	$\mathrm{\AA}_{2}>\mathrm{\AA}_{4}>\mathrm{\AA}_{5}>\mathrm{\AA}_{3}>\mathrm{\AA}_{1}$
${{\varvec{\lambda}}}_{8}=0.8$	0.81	0.98	0.84	0.92	0.88	$\mathrm{\AA}_{2}>\mathrm{\AA}_{4}>\mathrm{\AA}_{5}>\mathrm{\AA}_{3}>\mathrm{\AA}_{1}$
${{\varvec{\lambda}}}_{9}=0.9$	0.82	0.98	0.84	0.92	0.88	$\mathrm{\AA}_{2}>\mathrm{\AA}_{4}>\mathrm{\AA}_{5}>\mathrm{\AA}_{3}>\mathrm{\AA}_{1}$

**Fig. 3:**
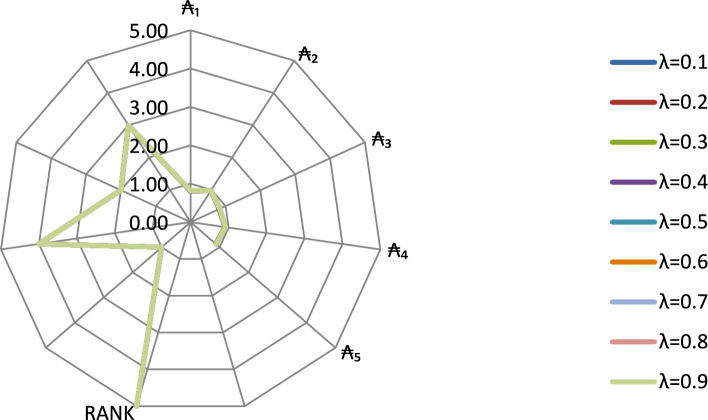
Geometrical representation of sensitivity analysis based on different parameters.

According to Table 16, the results show that the alternative $\mathrm{\AA}_{2}$ (E-Governance and Digital Policy Reform) has the highest mean score among all alternatives. This observation is fully consistent with the CRITIC–WASPAS ranking, which also identifies $\mathrm{\AA}_{2}$ as the most effective strategy for digital economic growth in each case of the parameters. The geometrical representation of sensitivity analysis is displayed in Figure 3.

### Practical implications

The CRITIC-WASPAS model, built on the CPFS framework, has strong practical value for organizations operating in the digital economy.

- Governments and policymakers can apply this framework to objectively prioritize digital economy strategies**,** such as infrastructure development, E-governance, and digital skills and education, etc., by accounting for both quantitative indicators and qualitative ℰ judgment under uncertainty.
- Real-world economic planning often involves conflicting stakeholder opinions**,** vague data, and hesitant assessments. The CPFS allows modeling of these uncertainties, ensuring that such indecisiveness is incorporated into the MAGDM process rather than ignored.
- The method is particularly suited to MAGDM**,** helping facilitate structured and transparent decision processes where ℰ from different domains contribute to complex evaluations.
- By integrating objective weighting with the help of the CRITIC method with robust aggregation by WASPAS, the framework promotes data-driven decision-making**,** reducing reliance on intuition or political bias in national digital transformation agendas.

## Result discussion and comparison

In the CPFS framework, where the uncertainty is extremely complicated, the suggested hybrid model can easily handle and integrate the aggregation technique. We summarized the comparative features of this paper in Table 17.

**Table 17:** Summary of comparative features.

Aspect	Uncertainty modeling	Attributes correlation	Directional data	Subjectivity level	Robustness in MADM
FS	Membership	No	No	Moderate	Moderate
IFS	Membership, non-membership, and indeterminacy	No	No	Moderate	Moderate
PFS	Membership, abstinence, non-membership, and refusal	No	No	Moderate	Moderate
CIFS	Membership, non-membership, indeterminacy, and directional	Yes	Yes	Low	High
CPFS	Membership, abstinence, non-membership, and refusal	Yes	Yes	High	Very High
Weighting: CRITIC	Uses correlation and contrast	Yes	N/A	Low	High
Weighting: Entropy	Data distribution only	No	N/A	Low	Moderate
Weighting: AHP	Subjective ℰ input	No	N/A	High	Low
Aggregation: WASPAS	Combines WSM and WPM	Indirectly via weights	N/A	Moderate	High
Aggregation: TOPSIS	Distance-based ranking	No	N/A	Moderate	Moderate
Aggregation: VIKOR	Compromise-based ranking	No	N/A	Moderate	Moderate

According to Table 17, we get the following results of the initiated work of this paper:

- Accurately captures uncertainty in expert opinions using CPFS.
- Objectively assigns importance to attributes with the CRITIC method.
- Reliably evaluates options using the WASPAS approach.

Compared to traditional methods like FS, IFS, PFS, CIFS, subjective weighting, and simple aggregation, this combined framework is more accurate, consistent, and flexible. This makes it more suitable for situations involving uncertainty and complex interactions between qualities in real-world decision-making.

### Compatibility and validation

To confirm the viability and superiority of the suggested hypothesis, a comparison study is carried out. The data in Table 4 are subjected to the suggested method, and the ranking outcomes are contrasted with those of previous approaches, such as those by Senapati and Gen^23^, Albaity et al.^24^, Xu^26^, Zhao et al.^27^, Fahmi et al.^28^, and Bozyigit et al.^29^. The comparison results, shown in Table 18, show the efficacy and validity of the suggested strategy.

**Table 18:** Comparison with existing literature.

Authors/Methods	Results and ranking	Best alternative
Senapati and Gen^23^	Table 4 data cannot be assessed	limited features
Albaity et al.^24^	Table 4 data cannot be assessed	limited features
Xu^26^	Table 4 data cannot be assessed	limited features
Zhao et al.^27^	Table 4 data cannot be assessed	limited features
Fahmi et al.^28^	Table 4 data cannot be assessed	limited features
Bozyigit et al.^29^	Table 4 data cannot be assessed	limited features
${{\varvec{Q}}}_{\mathcal{i}}^{1}$	$\mathrm{\AA}_{2}>\mathrm{\AA}_{4}>\mathrm{\AA}_{5}>\mathrm{\AA}_{3}>\mathrm{\AA}_{1}$	$\mathrm{\AA}_{2}$
${{\varvec{Q}}}_{\mathcal{i}}^{2}$	$\mathrm{\AA}_{2}>\mathrm{\AA}_{4}>\mathrm{\AA}_{5}>\mathrm{\AA}_{3}>\mathrm{\AA}_{1}$	$\mathrm{\AA}_{2}$
${{\varvec{Q}}}_{\mathcal{i}}$	$\mathrm{\AA}_{2}>\mathrm{\AA}_{4}>\mathrm{\AA}_{5}>\mathrm{\AA}_{3}>\mathrm{\AA}_{1}$	$\mathrm{\AA}_{2}$

Table 18 shows that the proposed approach is feasible and powerful due to its advanced features, whereas existing techniques are limited and unable to solve the data presented in Table 4. The main limitation of existing methods is their restricted frameworks, such as IFS, PyFS, and CPFS, which are based on algebraic properties, methods, generalizations, and operators. Unlike existing methods, the proposed approach operates on the CPF information, which provides a more comprehensive framework with a larger domain and circular behavior. This makes existing techniques a special case of the proposed work, highlighting the limitations of earlier established work.

## Conclusion

In order to assess methods for promoting economic growth in the digital economy, this study presents a new framework for decision-making that integrates the CRITIC and WASPAS approaches within the CPFS system. The framework can effectively convey hesitation, neutrality, and divergent viewpoints by employing circular fuzzy logic to more effectively convey uncertainty. While WASPAS offers a fair method of ranking options using both WSM and WPM, the CRITIC approach offers objective weights for each attribute. The model demonstrated efficacy in ranking digital-economy strategies under uncertain, multi-attribute conditions when used in a real case study. Further evidence that the model is reliable, consistent, and outperforms conventional fuzzy decision-making techniques came from sensitivity and comparison tests.

### Constraints and limitations

The CPF framework is more expressive and capable than traditional fuzzy set models. Within this framework, the extended CRITIC-WASPAS model offers a reliable and efficient solution for complex decision-making under uncertainty. However, its use is limited to situations where the data follows the structure defined in Definition 3. It currently does not support bipolar fuzzy information or circular complex fuzzy sets. Despite these limitations, the model significantly advances fuzzy decision-making and performs well across many practical applications.

### Future direction

The proposed work of this manuscript can be extended in future research according to other aggregation operators, such as power aggregation operators, Aczel-Alsina aggregation operators, and Dombi aggregation operators. Further, future research may also explore integrating other techniques like DEMATAL, MARCOS, and EDAS methods into the proposed framework. However, the concept can offer possible avenues for further research, like Aczel–Alsina power aggregation operator^30^, Dombi hamy mean operators^31^, TOPSIS method for MAGDM^32^, and Aczel-Alsina hamy mean operators^33^.

## Data Availability

The datasets used and/or analyzed during the current study are available from the corresponding author upon reasonable request.

## References

[1] Zadeh, L. A. Fuzzy sets. *Inf. Control* **8**(3), 338–353. 10.1016/S0019-9958(65)90241-X (1965).

[2] Atanassov, K. T. Intuitionistic fuzzy sets. *Fuzzy Sets Syst.* **20**(1), 87–96. 10.1016/S0165-0114(86)80034-3 (1986).

[3] B. C. Cuong, Picture fuzzy sets-first results. part 1, seminar neuro-fuzzy systems with applications *Inst. Math. Hanoi* (2013).

[4] Atanassov, K. T. Circular intuitionistic fuzzy sets. *J. Intell. Fuzzy Syst.* **39**(5), 5981–5986 (2020).

[5] Xu, Y. & Zhang, D. Identifying AI-driven emerging trends in service innovation and digitalized industries using the circular picture fuzzy WASPAS approach. *Symmetry* **17**(9), 1546 (2025).

[6] Liu, S. & Zhao, D. Diffusion and economic growth fuzzy intelligent system based on DSGE model. *J. Intell. Fuzzy Syst.* **40**(4), 5975–5983. 10.3233/JIFS-189437 (2021).

[7] F. Subkhan, M. S. Maarif, N. T. Rochman, and Y. Nugraha, Digital economy: reinforcing competitive economy of smart cities, A Fuzzy-AHP Approach In *2024 International Conference on ICT for Smart Society (ICISS)* IEEE 1–10 Accessed Oct 01 2025. Available: https://ieeexplore.ieee.org/abstract/document/10751367/ (2024)

[8] R. Imamguluyev, A. Panahov, A. Jabbarov, A. Hajiyev, and K. Aghayeva, “The Role of Fuzzy Logic in the Digital Transformation of Economics: Innovative Analysis and Strategies,” in *Intelligent and Fuzzy Systems*, vol. 1530, C. Kahraman, S. Cebi, B. Oztaysi, S. Cevik Onar, C. Tolga, I. Ucal Sari, and I. Otay, Eds., in Lecture Notes in Networks and Systems, vol. 1530. , Cham: Springer Nature Switzerland, 2025, pp. 676–683. 10.1007/978-3-031-98565-2_73.

[9] Zhang, X. & Wang, A. Enhancing the performance of vocational education in the digital economy with the application of fuzzy logic algorithm. *Int. J. Comput. Intell. Syst.* **17**(1), 185. 10.1007/s44196-024-00591-9 (2024).

[10] Kaur, P., Verma, R. & Mahanti, N. C. Selection of vendor using analytical hierarchy process based on fuzzy preference programming. *Opsearch* **47**(1), 16–34 (2010).

[11] Kaur, P. Selection of vendor based on intuitionistic fuzzy analytical hierarchy process. *Adv. Oper. Res.* **2014**, 1–10. 10.1155/2014/987690 (2014).

[12] Hussain, A., Ullah, K., Yang, M.-S. & Pamucar, D. Aczel-Alsina aggregation operators on T-spherical fuzzy (TSF) information with application to TSF multi-attribute decision making. *Ieee Access* **10**, 26011–26023 (2022).

[13] Wang, R., Wang, J., Gao, H. & Wei, G. Methods for MADM with picture fuzzy muirhead mean operators and their application for evaluating the financial investment risk. *Symmetry* **11**(1), 6 (2018).

[14] Kaur, P., Dutta, V., Pradhan, B. L., Haldar, S. & Chauhan, S. A pythagorean fuzzy approach for sustainable supplier selection using TODIM. *Math. Probl. Eng.* **2021**, 1–11. 10.1155/2021/4254894 (2021).

[15] P. Kaur and A. Priya, Selection of inventory policy under pythogrean fuzzy environment *Sci. Technol. Asia *62–71 (2020).

[16] M. R. Seikh and U. Mandal, Some picture fuzzy aggregation operators based on frank t-norm and t-conorm: application to MADM process *Informatica* 45(3):10.31449/inf.v45i3.3025 (2021)

[17] Hussain, A., Mahmood, T., Smarandache, F. & Ashraf, S. TOPSIS approach for MCGDM based on intuitionistic fuzzy rough Dombi aggregation operations. *Comput. Appl. Math.* **42**(4), 176. 10.1007/s40314-023-02266-1 (2023).

[18] Abbas, F., Ali, J. & Mashwani, W. K. Partitioned Hamy mean aggregation for multi-criteria group decision-making in the MAIRCA framework with q-rung orthopair fuzzy 2-tuple linguistic information. *Granul. Comput.* **9**(3), 62 (2024).

[19] Wang, H. Sustainable circular supplier selection in the power battery industry using a linguistic T-spherical fuzzy MAGDM model based on the improved ARAS method. *Sustainability* **14**(13), 7816 (2022).

[20] Zavadskas, E. K., Turskis, Z., Antucheviciene, J. & Zakarevicius, A. Optimization of weighted aggregated sum product assessment. *Elektron. Ir Elektrotechnika* **122**(6), 3–6 (2012).

[21] Rani, P., Mishra, A. R. & Pardasani, K. R. A novel WASPAS approach for multi-criteria physician selection problem with intuitionistic fuzzy type-2 sets. *Soft Comput.* **24**(3), 2355–2367 (2020).

[22] Ayyildiz, E., Erdogan, M. & Taskin Gumus, A. A Pythagorean fuzzy number-based integration of AHP and WASPAS methods for refugee camp location selection problem: a real case study for Istanbul, Turkey. *Neural Comput. Appl.* **33**(22), 15751–15768. 10.1007/s00521-021-06195-0 (2021).

[23] Senapati, T. & Chen, G. Picture fuzzy WASPAS technique and its application in multi-criteria decision-making. *Soft Comput.* **26**(9), 4413–4421. 10.1007/s00500-022-06835-0 (2022).

[24] Albaity, M., Mahmood, T. & Ali, Z. Impact of machine learning and artificial intelligence in business based on intuitionistic fuzzy soft WASPAS method. *Mathematics* **11**(6), 1453 (2023).

[25] Abbas, F., Ali, J., Mashwani, W. K., Gündüz, N. & Syam, M. I. q-Rung orthopair fuzzy 2-tuple linguistic WASPAS algorithm for patients’ prioritization based on prioritized Maclaurin symmetric mean aggregation operators. *Sci. Rep.* **14**(1), 10659 (2024). 38724560 10.1038/s41598-024-57452-w PMC11538463

[26] Xu, Z. Intuitionistic fuzzy aggregation operators. *IEEE Trans. Fuzzy Syst.* **15**(6), 1179–1187 (2007).

[27] Zhao, H., Xu, Z., Ni, M. & Liu, S. Generalized aggregation operators for intuitionistic fuzzy sets. *Int. J. Intell. Syst.* **25**(1), 1–30. 10.1002/int.20386 (2010).

[28] Fahmi, A., Khan, A., Maqbool, Z. & Abdeljawad, T. Circular intuitionistic fuzzy Hamacher aggregation operators for multi-attribute decision-making. *Sci. Rep.* **15**(1), 5618 (2025). 39955333 10.1038/s41598-025-88845-0 PMC11830057

[29] Bozyigit, M. C., Olgun, M. & Ünver, M. Circular pythagorean fuzzy sets and applications to multi-criteria decision making. *Informatica* **34**(4), 713–742. 10.15388/23-INFOR529 (2023).

[30] Mahmood, T. & Ali, Z. Multi-attribute decision-making methods based on Aczel-Alsina power aggregation operators for managing complex intuitionistic fuzzy sets. *Comput. Appl. Math.* **42**(2), 87. 10.1007/s40314-023-02204-1 (2023).

[31] Hussain, A., Ullah, K., Al-Quran, A. & Garg, H. Some T-spherical fuzzy dombi hamy mean operators and their applications to multi-criteria group decision-making process. *J. Intell. Fuzzy Syst.* **45**(6), 9621–9641 (2023).

[32] M. Riaz, K. Naeem, and D. Afzal, Pythagorean m-polar fuzzy soft sets with TOPSIS method for MCGDM *Punjab Univ. J. Math.* 52 (3) Accessed Feb 25 2025 Available http://journals.pu.edu.pk/journals/index.php/pujm/article/viewArticle/3469 (2020)

[33] Hussain, A., Wang, H., Ullah, K. & Pamucar, D. Novel intuitionistic fuzzy Aczel Alsina Hamy mean operators and their applications in the assessment of construction material. *Complex Intell. Syst.* **10**(1), 1061–1086. 10.1007/s40747-023-01116-1 (2024).

